# The possible role of the Microbiota-Gut-Brain-Axis in the etiology of Major depressive Disorder (MDD) The possible role of the Microbiota-Gut-Brain-Axis in the etiology of Major depressive Disorder (MDD) The possible role of the Microbiota-Gut-Brain-Axis in the etiology of Major depressive Disorder (MDD)

**DOI:** 10.1192/j.eurpsy.2022.1453

**Published:** 2022-09-01

**Authors:** T. Pieters, T. Van Der Gronde, L. Van Der Velde

**Affiliations:** Utrecht University, Pharmaceutical Sciences, Utrecht, Netherlands

**Keywords:** HPA axis, pro-inflammatory state, microbiota, MDD

## Abstract

**Introduction:**

1.Introduction MDD is a heterogeneous disorder, with a wide variety of symptoms and inconsistent treatment response, and is not completely understood. A dysregulated stress system is a consistent finding, however, and exhaustion is a consistent trait in adolescent patients. In order to open up our thinking about MDD we take up the challenge to reframe depression, specifically focusing on the possible role of the Microbiota-Gut-Brain-Axis in the etiology of MDD.

**Objectives:**

We propose a ‘bidirectional feedback hypothesis’: microbiota can promote or inhibit a pro-inflammatory state, (in)directly altering the hypothalamic pituitary adrenal (HPA) axis response and the microbiome and further increasing or decreasing its pro-inflammatory state. The aim is to show that the pro-inflammatory state is an integral part of a HPA axis stress spiralling mechanism that plays a role in the etiology of MDD.

**Methods:**

A systematic review based on publications from PubMed, Embase and PsycInfo

**Results:**

The etiology of MDD can be understood as sliding down a spiral. This stress spiralling mechanism can be promoted or inhibited by: 1.factors such as a poor lifestyle or (pre-existing) illness 2.bettering someone’s lifestyle, coping behavior or providing pro-/prebiotics in combination with personalised therapeutics.

**Conclusions:**

We argue that an interdisciplinary One Health approach is the most promising preventive and therapeutic option for MDD.

**Disclosure:**

No significant relationships.

